# Supplementary material for: Super-enhancer-based identification of a BATF3/IL-2R−module reveals vulnerabilities in anaplastic large cell lymphoma
Source: Nat Commun. 2021 Sep 22;12:5577. doi: 10.1038/s41467-021-25379-9 (PMC8458384; doi:10.1038/s41467-021-25379-9)
Supplement: Supplementary file 5 — Reporting Summary [file 41467_2021_25379_MOESM5_ESM.pdf]

nature research

Corresponding author(s): Olaf Merkel, Lukas Kenner, Stephan Mathas

Last updated by author(s): 10.June 2021

Reporting Summary

Nature Research wishes to improve the reproducibility of the work that we publish. This form provides structure for consistency and transparency in reporting. For further information on Nature Research policies, see our Editorial Policies and the Editorial Policy Checklist.

Statistics

For all statistical analyses, confirm that the following items are present in the figure legend, table legend, main text, or Methods section.

n/a	Confirmed	
☐	☒	The exact sample size (*n*) for each experimental group/condition, given as a discrete number and unit of measurement
☐	☒	A statement on whether measurements were taken from distinct samples or whether the same sample was measured repeatedly
☐	☒	The statistical test(s) used AND whether they are one- or two-sided<br>*Only common tests should be described solely by name; describe more complex techniques in the Methods section.*
☐	☒	A description of all covariates tested
☐	☒	A description of any assumptions or corrections, such as tests of normality and adjustment for multiple comparisons
☐	☒	A full description of the statistical parameters including central tendency (e.g. means) or other basic estimates (e.g. regression coefficient) AND variation (e.g. standard deviation) or associated estimates of uncertainty (e.g. confidence intervals)
☐	☒	For null hypothesis testing, the test statistic (e.g. *F*, *t*, *r*) with confidence intervals, effect sizes, degrees of freedom and *P* value noted<br>*Give P values as exact values whenever suitable.*
☒	☐	For Bayesian analysis, information on the choice of priors and Markov chain Monte Carlo settings
☐	☒	For hierarchical and complex designs, identification of the appropriate level for tests and full reporting of outcomes
☐	☒	Estimates of effect sizes (e.g. Cohen's *d*, Pearson's *r*), indicating how they were calculated

Our web collection on statistics for biologists contains articles on many of the points above.

Software and code

Policy information about availability of computer code

Data collection	E-CRISP program, version 5.4; super-enhancer assignment "ROSE" algorithm, version 2018; promoters were determined using bamToGFF (https://github.com/BradnerLab/pipeline); LASAGNA-Search 2.0 online tool. Computer code used in this study is available within Supplementary Software.
Data analysis	Ingenuity pathway analysis, version 2018 (Quiagen); for statistical analysis, Prism 6.0 for MacOSX and R v4.0.3 software were used; for calculation of Kaplan-Meier curves, IBM SPSS Statistics Version 22 with the survival package of R (v3.2-7; Therneau, 2020) were used; FACSDiva software 8.0.1 (Becton Dickinson).

For manuscripts utilizing custom algorithms or software that are central to the research but not yet described in published literature, software must be made available to editors and reviewers. We strongly encourage code deposition in a community repository (e.g. GitHub). See the Nature Research guidelines for submitting code & software for further information.

Data

Policy information about availability of data

All manuscripts must include a data availability statement. This statement should provide the following information, where applicable:

- Accession codes, unique identifiers, or web links for publicly available datasets
- A list of figures that have associated raw data
- A description of any restrictions on data availability

All RNA-seq and ChIP-seq datasets produced in this study are deposited in Gene Expression Omnibus (GEO) under SuperSeries GSE158916. Source data is provided for the Figures 1a-d, Supplementary Figure 1 and Supplementary Figure 2a-d. The Crescenzo et al. and Iqbal et al. publicly available data used in this study are available in the Sequence Read Archive (SRA) and GEO database under accession code SRP044708 and GSE19069, respectively. ChIP-seq of H3K27ac and input DNA of normal T-cell subsets and Jurkat cell line used in this study are publicly available in GEO under the accession numbers GSM1058764, GSM1058789, GSM772835,

GSM772916, GSM1102781, GSM1102806, GSM1296384 and GSM569086. The remaining data are available within the Article, Supplementary Information or Source Data file.

Field-specific reporting

Please select the one below that is the best fit for your research. If you are not sure, read the appropriate sections before making your selection.

- [x] Life sciences
- [ ] Behavioural & social sciences
- [ ] Ecological, evolutionary & environmental sciences

For a reference copy of the document with all sections, see nature.com/documents/nr-reporting-summary-flat.pdf

Life sciences study design

All studies must disclose on these points even when the disclosure is negative.

Sample size	ALCL is a rare disease; therefore, sample sizes above 10 are rarely seen in publications. To find BATF3-correlated genes, we used a previously published RNA-seq dataset of 23 ALCL patients (Crescenzo et al., Cancer Cell 2015) and microarray of 29 ALCL patients (Iqbal et al., Blood, 2010). For IHC staining, we used reactive lymph node controls (n = 11), AITL (n = 8), PTCL-NOS (n = 23), ALCL, ALK+ (n = 22), ALCL, ALK– (n = 23) and pcALCL (n = 24). These numbers are sufficient to provide reliable mean expression levels of the relevant proteins by IHC and all data points are shown to give a view on data distribution. For Kaplan-Meier analysis in Fig. 4 and Supplementary Fig. 6, we had 88 pediatric ALCL, ALK+ patients and 44 (34 without SCT and 10 with SCT) adult ALCL, ALK- patients. Kaplan-Meier analysis is used here to substantiate our claim that IL-2Rα has biological importance in ALCL, which is supported by many other functional experiments in this study. Since ALCL is a very rare disease, numbers reached in Kaplan-Meier analysis were only possible through Europe-wide collaboration and are sufficient to support the functional claims shown by other experiments. Moreover, two independent cohorts show similar results.
Data exclusions	In the IHC from Fig. 1h, Supplementary Fig 3b and 5c, data were excluded if the FFPE core in the particular tissue microarray was of low quality or absent, explaining the slight number differences in the cohorts. The pre-established exclusion criteria in pathology are absent tissue core or strongly damaged tissue core.
Replication	All in vitro experiments were performed with a minimum of three biological replicates. IHC analysis of proteins of interest were assessed in tissue microarrays containing primary materials from 3 independent cohorts with similar results. RNA-seq experiments were performed in 3 biological replicates and the mean of those experiments is used. All attempted replications did show similar results in RNA-seq and in vitro experiments and no outliers were observed.
Randomization	Mice: after tumor engraftment, mice were randomized into three groups with similar mean tumor size. Mice were treated with antibody control, PBS or armed antibody.
Blinding	Men: for IHC analysis of patient tumor samples, pathologists were blinded to group allocation and diagnosis.<br>Mice: due to small sample number and stark differences in tumor size, blinding to group allocation was not possible.

Reporting for specific materials, systems and methods

We require information from authors about some types of materials, experimental systems and methods used in many studies. Here, indicate whether each material, system or method listed is relevant to your study. If you are not sure if a list item applies to your research, read the appropriate section before selecting a response.

Materials & experimental systems

n/a	Involved in the study	
☐	☒	Antibodies
☐	☒	Eukaryotic cell lines
☒	☐	Palaeontology and archaeology
☐	☒	Animals and other organisms
☐	☒	Human research participants
☐	☒	Clinical data
☒	☐	Dual use research of concern

Methods

n/a	Involved in the study	
☐	☒	ChIP-seq
☐	☒	Flow cytometry
☒	☐	MRI-based neuroimaging

Antibodies

Antibodies used	ChIP and ChIP-seq:<br>Name/Catalogue No./Company<br>anti-BATF3/AF7437/R&D Systems<br>anti-H3K27ac/ab4729/Abcam<br><br>IHC:<br>Name/Catalogue No./Company

anti-CD30/M0751/Dako
anti-IL-2/sc-7896/Santa Cruz
anti-IL-2Rα/125M-16/Cell Marque
anti-IL-2Rβ/ab197934/Abcam
anti-IL-2Rγ/PA5-26461/Invitrogen
anti-BATF3/AF7437/R&D Systems
anti-IL-15/MAB2471/R&D Systems
anti-IL-15Rα/AF247/R&D Systems

Flow Cytometry:
Name/Catalogue No./Company
anti-IL-2Rα/12-0259-42/Invitrogen
anti-IL-2Rβ/11-1228-42/Invitrogen
anti-IL-2Rγ/PA5-26461/Invitrogen
anti-CD30/11-0309-41/Invitrogen
anti-IL-15Rα/AF247/R&D Systems
Isotype control/12-4714-82/Invitrogen
Isotype control/11-4714-82/Invitrogen
Isotype control/02-6102/Invitrogen
Isotype control/AB-108-C/R&D Systems

Immunoblotting:
Name/Catalogue No./Company
anti-BATF3/AF7437/R&D Systems
anti-STAT1/9172/Cell Signaling
anti-pSTAT1 (Y701)/9167/Cell Signaling
anti-STAT3/12640/Cell Signaling
anti-pSTAT3 (Y705)/9131/Cell Signaling
anti-STAT5/In-house
anti-pSTAT5 (Y694)/71-6900/Invitrogen
anti-ERK1/2/4695/Cell Signaling
anti-pERK1/2 (T202/Y204)/4370/Cell Signaling
anti-PARP1/9532/Cell Signaling
anti-β-ACTIN/4967/Cell Signaling
anti-β-TUBULIN/2146/Cell Signaling
anti-GAPDH/2275-PC-100/Trevigen

Validation

If not validated by the manufacturer, we validated every antibodies with positive (tissue/cell line with confirmed expression) and negative control (genetic knockouts or tissue with confirmed absence of expression).

Eukaryotic cell lines

Policy information about cell lines

Cell line source(s)

If not stated otherwise, ALCL cell lines were obtained from the ATCC cell line repository. Non-ALCL T-cell leukemia-derived cell lines were received from the DSMZ collection (Braunschweig, Germany). Cell lines are regularly tested for mycoplasma contamination, and cell line identity has been verified by STR DNA fingerprinting (R. Siebert, Ulm, Germany). BIA-ALCL (TLBR-1, TLBR-2 and TLBR-3) cell lines were kindly provided by Alan Epstein (California, USA) and cultured in RPMI 1640 supplemented with 10% FBS, 100 IU/ml penicillin, 50 mg/ml streptomycin sulfate and 330 ng/ml in-house synthesized IL-2-Fc. FE-PD cell line was kindly provided by Annarosa del Mistro as a gift (Veneto Onocology Insitute Padua, Italy). Mac-1 and Mac-2A cell line were kindly provided by Marshall E. Kadin as a gift (Harvard Medical School, Boston, MA). DL-40 was kindly provided by Masanori Daibata (Kochi, Japan).

Authentication

Microsatellite authentication has been performed with systemic ALCL cell lines in the Department of Medical Genetics in Ulm by Prof. Siebert in Germany.

Mycoplasma contamination

As a regular standard procedure in our laboratory, all cell lines were tested for mycoplasma contamination after thawing and prior to use in the experiments described.

Commonly misidentified lines
(See ICLAC register)

None of those have been used.

Animals and other organisms

Policy information about studies involving animals; ARRIVE guidelines recommended for reporting animal research

Laboratory animals

Immunocompromised NOD.Cg-PrkdcscidIl2rgtm1Wjl/SzJ (NSG) recipients were purchased (Jackson Laboratory, USA).

Wild animals

No wild animals was used in this study.

Field-collected samples	No field-collected samples was used in this study.
Ethics oversight	License number approved by the Austrian Ministry for Science and Research: Role of TF BATF and BATF3 in ALCL BMWF-66.009/0375-V/3b/2019 and BMWF-66.009/0391-V/3b/2019.

Note that full information on the approval of the study protocol must also be provided in the manuscript.

Human research participants

Policy information about studies involving human research participants

Population characteristics	Population characteristics such as age, gender and diagnosis of patients used for this study are provided in Supplementary Table 3 and 6.
Recruitment	Patient tissues used for IHC were derived from published, well-annotated clinical trial cohorts. The ALCL99 trial launched by the European Intergroup for Childhood Non-Hodgkin's Lymphoma between November 1999 and June 2006. FFPE (formalin-fixed paraffin embedded) tissues from systemic ALCL, ALK- cases were collected through TENOMIC, a transnational research consortium on T-cell lymphomas involving several centers in France, Belgium and Switzerland. Clinical data for the Brno and TENOMIC patients are given in Supplementary Table 6.
Ethics oversight	All human samples were obtained with informed patient consent and in accordance with the Declaration of Helsinki reviewed by the local ethic boards (Medical University of Vienna 1221/2019; University Hospital Brno 4-306/13/1). FFPE tissues from systemic ALCL, ALK- cases collected through TENOMIC, a transnational research consortium on T-cell lymphomas involving several centers in France, Belgium and Switzerland, were used for immunohistochemical validation. The TENOMIC database is approved by the ethical committee "CPP Ile-de-France IX 08-009".

Note that full information on the approval of the study protocol must also be provided in the manuscript.

Clinical data

Policy information about clinical studies

All manuscripts should comply with the ICMJEguidelines for publication of clinical research and a completedCONSORT checklist must be included with all submissions.

Clinical trial registration	The work submitted does not represent a clinical trial. However, we used tissue samples from patients treated in the ALCL99 trial and TENOMIC (see ref. Acc. No. 22084369, 21280197, 12635465 and 11389005).
Study protocol	The work submitted does not represent a clinical trial. However, we used tissue samples from patients treated in the ALCL99 trial and TENOMIC (see ref. Acc. No. 22084369, 21280197, 12635465 and 11389005).
Data collection	Patient tissues used for IHC were derived from published, well-annotated clinical trial cohorts. The ALCL99 trial launched by the European Intergroup for Childhood Non-Hodgkin's Lymphoma between November 1999 and June 2006. FFPE tissues from systemic ALCL, ALK- cases were collected through TENOMIC, a transnational research consortium on T-cell lymphomas involving several centers in France, Belgium and Switzerland. Clinical data for the Brno and TENOMIC patients are given in Supplementary Table 6.
Outcomes	IHC results were linked to clinical data from published clinical studies. The primary objective of the ALCL99 trial was to estimate the differences in the event-free survival at 2 years of patients treated in two Methotrexate administration arms (difference: Methotrexate dose and infusion time). Events were defined as progression or relapse, second malignancy or death of any cause. Secondary outcomes were overall survival, complete remission, CNS-relapse and acute toxicity. Events and toxicity were assessed on the respective case report forms from the study (paper-CRF). The status of the patients was queried by the national study center every year.

ChIP-seq

Data deposition

☒ Confirm that both raw and final processed data have been deposited in a public database such as GEO.

☒ Confirm that you have deposited or provided access to graph files (e.g. BED files) for the called peaks.

Data access links<br>*May remain private before publication.*	https://www.ncbi.nlm.nih.gov/geo/query/acc.cgi?acc=GSE158916
Files in database submission	GSM4815739, ALK+ ALCL_Karpas-299_Input<br>GSM4815740, ALK+ ALCL_Karpas-299_H3K27ac<br>GSM4815741, ALK+ ALCL_Karpas-299_BATF3<br>GSM4815742, ALK– ALCL_Mac-1_Input<br>GSM4815743, ALK– ALCL_Mac-1_H3K27ac<br>GSM4815744, ALK– ALCL_Mac-1_BATF3<br>GSM5277975, ALK+ ALCL_JB6_Input<br>GSM5277976, ALK+ ALCL_JB6_H3K27ac<br>GSM5277977, ALK+ ALCL_SUP-M2_Input<br>GSM5277978, ALK+ ALCL_SUP-M2_H3K27ac

GSM5277979, ALK+ ALCL_SR-786_Input
GSM5277980, ALK+ ALCL_SR-786_H3K27ac
GSM5277981, ALK+ ALCL_SU-DHL-1_Input
GSM5277982, ALK+ ALCL_SU-D-HL1_H3K27ac
GSM5277983, ALK– ALCL_FE-PD_Input
GSM5277984, ALK– ALCL_FE-PD_H3K27ac
GSM5277985, ALK– ALCL_Mac-2A_Input
GSM5277986, ALK– ALCL_Mac-2A_H3K27ac
GSM5277987, ALCL Primary #54_Input
GSM5277988, ALCL Primary #54_H3K27ac
GSM5277989, ALCL Primary #208_Input
GSM5277990, ALCL Primary #208_H3K27ac

Genome browser session (e.g. UCSC)

No longer applicable.

Methodology

Replicates

ChIP-seq was performed in 8 independent ALCL cell lines and 2 primary patient samples using antibodies for BATF3 (AF7437, R&D Systems) and H3K27ac (ab4729, Abcam).

Sequencing depth

	Sequenced	Aligned
K299_H3K27ac_MWZ7126_nm_treat_afterfiting_all.RPM.wig	49201366	43178903
K299_BATF3_MWZ7126_nm_treat_afterfiting_all.RPM.wig	40007874	34282333
K299_Input_MWZ7126_nm_treat_afterfiting_all.RPM.wig	45774596	39377762
MAC1_H3K27ac_MWZ7126_nm_treat_afterfiting_all.RPM.wig	49983050	43980860
MAC1_BATF3_MWZ7126_nm_treat_afterfiting_all.RPM.wig	42460152	36115750
MAC1_Input_MWZ7126_nm_treat_afterfiting_all.RPM.wig	49672814	42472956

Antibodies

Name/Catalogue No./Company
anti-BATF3/AF7437/R&D Systems
anti-H3K27ac/ab4729/Abcam

Peak calling parameters

Reads were aligned to the human genome (hg19) using bowtie with parameters –k 2 –m 2 [removed e 70] –best and –l set to the read length. For visualization, WIG files were created from aligned ChIP-seq read positions using MACS with parameters –w –S –space=50 –nomodel –shiftsize=200 to artificially extend reads to be 200bp and to calculate their density in 50bp bins. Read counts in 50bp bins were then normalized to the millions of mapped reads, giving reads per million (rpm) values. Regions enriched in ChIP-seq signal were identified using MACS with corresponding control and parameters –keep-dup=auto and –p 1e-9.

Data quality

Data quality was assessed using MACS by comparing peak enrichment over input controls with a p cutoff value of 1e-9

Software

Reads were aligned to the human genome (hg19) using bowtie with parameters –k 2 –m 2 –e 70 –best and –l set to the read length.

For visualization, WIG files were created from aligned ChIP-Seq read positions using MACS with parameters –w –S –space=50 –nomodel –shiftsize=200 to artificially extend reads to be 200bp and to calculate their density in 50bp bins.

Super-enhancers were identified using ROSE (https://bitbucket.org/young_computation/rose), as described in Mansour et al. with some modifications. Briefly, two sets of peaks of H3K27ac were identified using MACS with parameter sets – keep-dup=auto –p 1e-9 and –keep-dup=all –p 1e-9.

Flow Cytometry

Plots

Confirm that:

- [x] The axis labels state the marker and fluorochrome used (e.g. CD4-FITC).
- [x] The axis scales are clearly visible. Include numbers along axes only for bottom left plot of group (a 'group' is an analysis of identical markers).
- [x] All plots are contour plots with outliers or pseudocolor plots.
- [x] A numerical value for number of cells or percentage (with statistics) is provided.

Methodology

Sample preparation

We used ALCL cell lines that were washed twice in PBS and then incubated with PI and AnnexinV in calcium-containing buffer, which were subjected to subsequent two additional washing steps before analysis with flow cytometry.

Instrument

Data were acquired with a BD FACSCanto II and analyzed with FACSDiva software (Becton Dickinson).

Software

Flow cytometric data were analyzed with FACSDiva software (Becton Dickinson) and with Flow Jo v10.7

Cell population abundance	Not applicable as we have not performed cell sorting.
Gating strategy	In forward and side scatters, we excluded debris to obtain intact cells. The percentage of cells that were positive for PI, Annexin V or both is given in the respective figures.

☒ Tick this box to confirm that a figure exemplifying the gating strategy is provided in the Supplementary Information.
