# Supplementary material for: Super-enhancer-based identification of a BATF3/IL-2R−module reveals vulnerabilities in anaplastic large cell lymphoma
Source: Nat Commun. 2021 Sep 22;12:5577. doi: 10.1038/s41467-021-25379-9 (PMC8458384; doi:10.1038/s41467-021-25379-9)
Supplement: Supplementary file 3 — Description of Additional Supplementary Files [file 41467_2021_25379_MOESM3_ESM.pdf]

Description of Additional Supplementary Files

File Name: Supplementary Software 1

Description: The document contains code used to call super-enhancers in sequencing results of ChIP with H3K27ac antibody in the cell lines ALCL Karpas-299, Mac-1, JB6, Mac-2A, SR-786, SU-DHL-1, SUP-M2, FE-PD and in primary ALCL samples. The following codes are listed: bamToGFF; normalize_WIG_to_RPM; ROSE_geneMapper; call_and_assign_enhancers; utils.
