# Supplementary material for: Super-enhancer-based identification of a BATF3/IL-2R−module reveals vulnerabilities in anaplastic large cell lymphoma
Source: Nat Commun. 2021 Sep 22;12:5577. doi: 10.1038/s41467-021-25379-9 (PMC8458384; doi:10.1038/s41467-021-25379-9)
Supplement: Supplementary file 1 — Supplementary Information [file 41467_2021_25379_MOESM1_ESM.pdf]

Supplementary Table 1. 225 genes associated with SEs in both Karpas-299 and Mac-1 ALCL cell lines.

BATF3	*IL2RB*	*IRF4*	*JUNB*	*STAT1*	*TNFRSF8/CD30*	*ACTN4*	*ADD3*	*ADRBK1*
AGXT2L2	*AHNAK*	*AKNA*	*ANKRD28*	*ANP32A*	*APOLD1*	*ARF6*	*ARID1A*	*ASB9P1*
ASXL1	*ATP6AP1L*	*ATP8B4*	*ATXN1*	*AZIN1*	*B4GALT5*	*BATF*	*BCL3*	*BCOR*
BHLHE40	*C10orf10*	*C12orf67*	*C13orf18*	*C14orf181*	*C14orf182*	*C14orf43*	*C19orf61*	*C1orf57*
C21orf91	*C22orf26*	*C5orf56*	*C6orf223*	*CELF2*	*CFLAR*	*CHAC2*	*CHORDC1*	*CHSY1*
CISH	*CLEC16A*	*CMAH*	*CORO1A*	*CPPED1*	*CSGALNACT2*	*CTSB*	*CYTIP*	*DAD1*
DDIT4	*DENND3*	*DUSP22*	*ELMO1*	*ENO1*	*ETV6*	*FAM129B*	*FAM167A*	*FAM83C*
FARS2	*FLJ21408*	*FLJ32224*	*FMNL1*	*FNDC3B*	*FOSL2*	*FURIN*	*GALM*	*GALNT10*
GPR15	*GPR183*	*GPR68*	*GPRIN3*	*GRLF1*	*GSDMD*	*GYG1*	*GZMB*	*HECA*
HIC1	*HIF1A*	*HIPK2*	*HLX*	*HNRNPF*	*IER2*	*IFITM1*	*IGF2R*	*IKZF1*
IL1R1	*IL1R2*	*IL1RAP*	*IL22*	*IL32*	*IL4R*	*INPP4A*	*IRF2*	*IRF2BP2*
IRF5	*ITFG3*	*ITPRIPL1*	*ITPRIPL2*	*KIAA0182*	*KIAA1949*	*KSR1*	*LAMB3*	*LARS2*
LEPROT	*LIMD1*	*LIMK2*	*LOC100129316*	*LOC150776*	*LOC200261*	*LOC338799*	*LOC400927*	*LOC442421*
LOC541471	*LOC648987*	*LY86-AS1*	*MALAT1*	*MAP7*	*MBNL1*	*MBP*	*MCL1*	*MED9*
MFHAS1	*MGLL*	*MIDN*	*MIR1208*	*MIR142*	*MIR21*	*MIR23A*	*MIR3922*	*MIR4269*
MIR922	*MLLT4*	*MYB*	*MYH9*	*MYO1G*	*MYOM2*	*NCALD*	*NCK2*	*NCRNA00152*
NCRNA00261	*NCS1*	*NEURL3*	*NFIL3*	*NOTCH2*	*NTRK1*	*NTRK2*	*OBFC2A*	*ODZ4*
OMA1	*PAG1*	*PDE4B*	*PDE4D*	*PGAP1*	*PIM3*	*PITPNC1*	*PKM2*	*PRCC*
PRDM1	*PRELID1*	*PREP*	*PSTPIP1*	*PTP4A2*	*PTPN3*	*PTPRJ*	*RALGDS*	*RAPGEF1*
RBM15	*RBMS1*	*RFFL*	*RFTN1*	*RIN3*	*RNF157*	*RNF213*	*RORA*	*RORC*
RUNX2	*RUNX3*	*S1PR1*	*SBNO2*	*SDHAP2*	*SH3TC1*	*SLA*	*SLC15A4*	*SLC38A6*
SLC7A5P1	*SMAD3*	*SMAP2*	*SMPD4*	*SNORA14B*	*SOCS1*	*SPATA2*	*SQRDL*	*SSH1*
ST3GAL1	*ST8SIA4*	*STARD13*	*TAB2*	*TBC1D2B*	*TMEM163*	*TMEM49*	*TNFAIP3*	*TNFAIP8*
TNS3	*TOX*	*TPM4*	*TRAF1*	*TRIO*	*TRPS1*	*TSHZ2*	*UBC*	*UBE2E1*
WDR67	*YWHAQ*	*ZC3H4*	*ZFP36*	*ZNF217*	*ZNF292*	*ZNF395*	*ZNF438*	*ZNRF1*

Supplementary Table 2. *BATF3*-KO deregulated genes with BATF3 ChIP-seq enrichment.

	Number of genes	Genes with BATF3 ChIP-seq enrichment (%)
**Downregulated genes in RNA-seq of *BATF3*-KO cells**	581	354 (61%)

	Number of genes	Genes with BATF3 ChIP-seq enrichment (%)
**Upregulated genes in RNA-seq of *BATF3*-KO cells**	865	368 (43%)

**Downregulated TFs with BATF3 ChIP-seq Enrichment (n = 30)**					
AHR	*BCL6*	*CREM*	*DDIT3*	*FOS*	*HLX*
ID2	*JUN*	*KLF10*	*MXD1*	*MYB*	*RBPJ*
SATB1	*SOX2*	*STAT1*	*TBX3*	*TFAM*	*TFEC*
TGIF1	*TSC22D1*	*TSHZ1*	*TSHZ3*	*VSX1*	*ZBED1*
ZFY	*ZNF180*	*ZNF267*	*ZNF34*	*ZNF496*	*ZNF680*

**Upregulated TFs with BATF3 ChIP-seq Enrichment (n = 21)**						
BHLHE40	*FOXQ1*	*GLI3*	*GSC*	*HLF*	*HOXB9*	*KLF4*
KLF5	*MSC*	*NFATC2*	*NFIB*	*NR1I2*	*ONECUT2*	*SOX5*
STAT4	*TCF7L1*	*TEAD4*	*ZBTB20*	*ZNF219*	*ZNF396*	*ZNF532*

Supplementary Table 3. Clinical information and histoscore of indicated proteins of FFPE mature T-NHL TMA.

Case	Sex	Age at diagnosis	Diagnosis	Tissue Origin	Histoscore					
					BATF3	IL-2Rα	IL-2Rβ	IL-2Rγ	IL-15Rα	IL-2
1	M	51-60	ALCL, ALK^{+}	Inguinal lymph node	0.3	0.05	1.5	2.1	0.07	0
2	M	11-20	ALCL, ALK^{+}	Cervical lymph node	0.93	2.3	1.9	2.6	0.12	0
3	F	11-20	ALCL, ALK^{+}	Cervical lymph node	1.4	2.4	1.5	2.4	0.3	
4	M	51-60	ALCL, ALK^{+}	Cervical lymph node	0.2	1.5	0.9	0.75	0.15	0
5	M	11-20	ALCL, ALK^{+}	Cervical lymph node	0.4	1.5	2	2.2	0.07	0
6	M	11-20	ALCL, ALK^{+}	Buccal mucosa	0.53	1.8	1.35	1.3	0.05	0
7	M	31-40	ALCL, ALK^{+}	Abdominal mass	0.4	1.5	1.8	1.8	0.03	0
8	M	31-40	ALCL, ALK^{+}	Axillary mass	1	2	1.5	0.5	0.01	0
9	M	11-20	ALCL, ALK^{+}	Paravertebral soft tissue	0.43	0.13	1.05	2	0.17	
10	M	0-10	ALCL, ALK^{+}	Cervical lymph node	0.53	1.5	1.5	2.3	0.18	
11	M	51-60	ALCL, ALK^{+}	Inguinal lymph node	1.4	2.7	1.9	2.1	0.23	0
12	F	11-20	ALCL, ALK^{+}	Neck swelling	1.4	2.7	1.05	1.95	0.2	0
13	F	61-70	ALCL, ALK^{+}	Mesenteric lymph node	0	0.07	2.1	2.25	0.1	0
14	M	31-40	ALCL, ALK^{+}	Cervical lymph node	0.7	2.7	2.3	2.2	0.13	0
15	M	21-30	ALCL, ALK^{+}	Mesenteric nodule and nodular anterioror part of stomach	0.2	1.5	1.2	1.2	0.01	
16	M	21-30	ALCL, ALK^{+}	Lymph node	0.2		0.3	0.3		
17	M	41-50	ALCL, ALK^{+}	Subhepatal infiltrate	1.3	2.7	1.6	1.6	0.3	
18	M	51-60	ALCL, ALK^{+}	Axillary lymph node	1.55	2.1	0.8	1	0.05	0
19	M	21-30	ALCL, ALK^{+}	Axillary lymph node	1.1	2.4	0.45	0.4	0.025	
20	M	21-30	ALCL, ALK^{+}	Lymph node	0.8	1.5	0.2	0.1	005	0
21	F	21-30	ALCL, ALK^{+}	Cervical lymph node	1.25	2.25	0.3	0.3		
22	F	N/A	ALCL, ALK^{+}	Skin	0.9	0.23	1.3	0.1	0	
23	F	21-30	ALCL, ALK^{-}	Supraclavicular lymph node	0.1	0.07	0.6	1.6	0.08	0
24	M	31-40	ALCL, ALK^{-}	Mesenteric lymph node	0	0	0.07	1.3	0.01	0
25	M	31-40	ALCL, ALK^{-}	Axillary lymph node	0.67	1.5	1.73	2.2	0.01	0
26	M	71-80	ALCL, ALK^{-}	Supraclavicular lymph node	0.15	0.3	1.12	0.65	0.01	0
27	M	21-30	ALCL, ALK^{-}	Skin	0.13	0.97	0.83	1.27	0.003	0
28	F	11-20	ALCL, ALK^{-}	Axillary lymph node	0.12	0.8	0.67	0.8	0.01	0
29	M	51-60	ALCL, ALK^{-}	Cervical lymph node	0.67	0.8	1.7	1.07	0.02	0
30	M	11-20	ALCL, ALK^{-}	Inguinal lymph node	1.1	0.07	0.9	1.1	0.003	0
31	F	81-90	ALCL, ALK^{-}	Cervical lymph node	0.45	0.38	0.65	1.1	0.01	0
32	M	71-80	ALCL, ALK^{-}	Inguinal lymph node	1.6	2.7	1.4	0.65	0.03	0
33	M	61-70	ALCL, ALK^{-}	Nasopharynx	1.1		0.3	0.18	0	0
34	M	51-60	ALCL, ALK^{-}	Inguinal lymph node	1.6	0	1.3	0.5	0	0
35	M	51-60	ALCL, ALK^{-}	Supraclavicular lymph node	1.2	2.7	1	1.1	0	0
36	F	51-60	ALCL, ALK^{-}	Lymph node	0.45	0.6	0.25	0.55	0.01	
37	M	51-60	ALCL, ALK^{-}	Inguinal lymph node	1.3	2.4	1.2	0.45		0
38	M	81-90	ALCL, ALK^{-}	Mandible	0.05	1.35	0.25	0.3	0.1	0
39	M	51-60	ALCL, ALK^{-}	Retroperitoneal lymph nodes	0.05	0.23	0.1	0.1	0	

40	M	51-60	ALCL, ALK^{-}	Inguinal lymph node	1.6	0.6	0.1	0.1		
41	F	41-50	ALCL, ALK^{-}	Mesentery and retroperitoneum with pancreas	0.65	0.05	0.1	0.4	0.02	0
42	F	51-60	ALCL, ALK^{-}	Supraclavicular lymph node	0	0.1	1.05	0.6	0.01	0
43	M	51-60	ALCL, ALK^{-}	Lymph node	1.1	0.1	0.3	0.3	0.02	0
44	M	61-70	ALCL, ALK^{-}	Cervical lymph node	1.6	0.6	0.1	0.5		0
45	M	61-70	ALCL, ALK^{-}	Cervical lymph node	0.4		0.2	0.2		0
46	F	51-60	AITL	Cervical lymph node	0	0.03	0.25	0.3		
47	M	61-70	AITL	Axillary lymph node	0	0.2	0.6	0.5	0	
48	F	51-60	AITL	N/A	0					
49	F	51-60	AITL	N/A	0	0.05	0.05	0.08	0	
50	M	51-60	AITL	Inguinal lymph node	0.05	0.2	0.1	0.3	0.01	
51	F	81-90	AITL	Lymph node	0.15	0.05	0.2	0.1	0.01	
52	M	51-60	AITL	Cervical lymph node	0.13	1.2	0.3	0.4	0	
53	F	81-90	AITL	Inguinal lymph node	0.01	0.2	0.2	0.2	0	
54	F	71-80	PTCL-NOS	Mesenteric lymph node	0.05	0.02	0.08	0.08	0.01	
55	M	N/A	PTCL-NOS	Skin	0.05					
56	M	71-80	PTCL-NOS	Supraclavicular lymph node	0	0.15	0.5	0.3	0	
57	M	71-80	PTCL-NOS	Inguinal lymph node	0	0.3				
58	F	61-70	PTCL-NOS	Supraclavicular lymph node	0	0.03	0.55	0.55	0	
59	F	61-70	PTCL-NOS	Axillary lymph node	0.15	0.4	1.3	0.4	0.15	
60	F	61-70	PTCL-NOS	Lymph node	0.03	0.1	0.11	0.15	0	
61	M	61-70	PTCL-NOS	Cervical lymph node	0.55	0.3	0.2	0.3	0.03	
62	F	61-70	PTCL-NOS	Peritoneum	0	0.1	0.11	0.3	0	
63	M	51-60	PTCL-NOS	Cervical lymph node	0.1					
64	M	51-60	PTCL-NOS	Inguinal lymph node	0	0	0	0.3	0	
65	M	51-60	PTCL-NOS	Supraclavicular lymph node	0.05	0.2	0.3	0.2	0.2	
66	F	71-80	PTCL-NOS	Skin	0	0.1	0.01	0.6	0.06	
67	F	81-90	PTCL-NOS	Skin	0	1.2	0.2	0.2	0	
68	F	81-90	PTCL-NOS	Skin	0	0.08	0.03	0	0	
69	M	71-80	PTCL-NOS	Preauricular lymph node	0.4	0.02	0.6	0.1	0.1	
70	M	61-70	PTCL-NOS	Axillary lymph node	0	0	0.05	0.08	0	
71	M	41-50	PTCL-NOS	Inguinal lymph node	0	0.1				
72	F	71-80	PTCL-NOS	Inguinal lymph node	0	1.2	0.2	0	0	
73	F	71-80	PTCL-NOS	Axillary lymph node	0	0.1	0.2	0.6	0.1	
74	F	61-70	PTCL-NOS	Inguinal lymph node	0	0.2	0.23	0.3	0	
75	M	51-60	PTCL-NOS	Inguinal lymph node	0.03	0	0.1	0.08	0	
76	F	71-80	PTCL-NOS	Inguinal lymph node	0	0.1	0.8	0.4	0.13	
77	N/A	N/A	Healthy donor	Reactive lymph node	0	0	0	0	0	
78	N/A	N/A	Healthy donor	Reactive lymph node	0	0	0	0	0	
79	N/A	N/A	Healthy donor	Reactive lymph node	0	0	0	0	0	
80	N/A	N/A	Healthy donor	Reactive lymph node	0	0.1	0.1	0.2	0	
81	N/A	N/A	Healthy donor	Reactive lymph node	0	0.1	0.1	0.2	0	
82	N/A	N/A	Healthy	Reactive lymph node	0	0.1	0	0	0	

			donor							
83	N/A	N/A	Healthy donor	Reactive lymph node	0	0.1	0	0	0	
84	N/A	N/A	Healthy donor	Reactive lymph node	0	0.1	0	0	0	
85	N/A	N/A	Healthy donor	Reactive lymph node	0	0.03	0.2	0.05	0	
86	N/A	N/A	Healthy donor	Reactive lymph node	0.03	0.05	0.15	0.06	0	
87	N/A	N/A	Healthy donor	Reactive lymph node	0	0.05	0.2	0.02	0	

Supplementary Table 4. Clinical information and histoscore of indicated proteins of FFPE pcALCL tissue specimens.

Case	Tissue Origin	Histoscore			
		BATF3	IL-2Rα	IL-2Rβ	IL-2Rγ
1	Skin, arm	0	0.005	0.35	1.8
2	Skin	0.55	2.7	1.65	1.3
3	Skin, shoulder	0	2.25	1.4	
4	Skin, thigh	0.5	2.7	1.3	
5	Skin, arm	0.05	0.3	0.15	
6	Skin, neck	0.05	1.5	1.4	
7	Skin		2.7		
8	Skin, thorax wall	0.01	2.4	0.01	
9	Skin, frontal area		1.4	0.8	
10	Skin, back	0.2			
11	Skin, leg	0.05	2.7	0.4	0.6
12	Skin, head	0	0	0.01	0.05
13	Skin, leg	0	0.05	1.8	1.8
14	Skin, leg	0.75	2.7	1.4	0.8
15	Skin, leg	0.01	0.5	0.1	1.2
16	Skin, scalp	0.1	2.7	0.6	1.6
17	Skin, knee	0	0.02	0.01	1.6
18	Skin, buttocks	0	0	1.4	1.6
19	Skin, leg	0.4	0.05	0.9	0.5
20	Skin, thigh	0.01	1.6	0.4	0.8
21	Skin, thigh	0	0.4	1.7	0.6
22	Skin, arm	0	0	0.25	0.15
23	Skin, back	0.1	0.05	0	1.2
24	Skin, buttocks	0.01	0.5	0	0.8

Supplementary Tables 5. IL-15 histoscore of fresh frozen primary ALCL tissue specimens.

Case	ALK Status	Staining Intensity	% of Positive Cells	Histoscore
1	ALK^{+}	1	5%	0.05
2	ALK^{+}	1	10%	0.1
3	ALK^{+}	1	5%	0.05
4	ALK^{+}	1	10%	0.1
5	ALK^{-}	2	25%	0.25
6	ALK^{-}	2	80%	1.6
7	ALK^{-}	3	90%	2.7
8	ALK^{-}	0	0	0
9	ALK^{-}	2	70%	1.4

Supplementary Tables 6. Clinical characteristics of adult ALCL, ALK⁻ patients.

Case	Sex	Age at diagnosis	Date of Diagnosis	Frontline Treatment	Consolidative SCT (No = 0)	Relapse (No = 0, Yes = 1)	Death (No = 0, Yes = 1)	IL-2Rα Expression (Low = 0, High = 1)
1	M	41-50	2002	ACVBP	0	0	1	1
2	M	41-50	2009	ACVBP	0	1	1	1
3	M	41-50	2005	CHEP	0	1	1	1
4	F	61-70	2017	CHOEP	0	1	1	0
5	F	61-70	2015	CHOEP	0	1	1	0
6	M	41-50	2007	CHOP	0	0	0	0
7	F	51-60	2018	CHOP	0	0.	0	0
8	M	71-80	2007	CHOP	0	1	1	0
9	M	61-70	2015	CHOP	0	1	0	1
10	M	71-80	2010	CHOP	0	1	1	0
11	F	51-60	2007	CHOP	0	1	1	0
12	M	81-90	2012	CHOP	0	0	0	0
13	M	51-60	2008	CHOP	0	0	0	1
14	M	61-70	2004	CHOP	0	0	1	1
15	M	41-50	2008	CHOP	0	1	0	0
16	M	61-70	2015	CHOP	0	1	0	0
17	M	51-60	2016	CHOP	0	1	0	1
18	F	61-70	2010	CHOP	0	0	0	1
19	M	41-50	2007	CHOP	0	1	1	0
20	M	81-90	2001	CVP	0	1	1	0
21	M	81-90	2014	Mini CHOP	0	0	0	0
22	F	71-80	2006	Mini CHOP	0	1	1	1
23	M	51-60	2015	CHOEP	0	0	0	0
24	M	51-60	2012	CHOEP	0	0.	0	1
25	M	51-60	2013	CHOP	0	0	0	1
26	F	51-60	2004	ABVD + CHOP	Auto	0	0	0
27	M	51-60	2007	CHOEP	Auto	0	0	0
28	M	31-40	2007	CHOEP	0	1	0	0
29	M	71-80	2014	CHOP	0	0	1	1
30	F	81-90	2014	CVP	0	0	1	1
31	M	51-60	2014	CHOP	0	1	1	1
32	F	41-50	2007	CHOP + ICE	0	1	1	1
33	M	61-70	2011	CHOEP	0	1	1	1
34	M	61-70	2011	CHOP	0	1	1	1
35	M	31-40	2009	ACVBP	Allo	0	1	0
36	M	51-60	2004	ACVBP	Auto	0	0	0
37	M	41-50	2006	ACVBP	Tandem auto-miniallo	0	0	1
38	M	51-60	2009	CHOP	Allo	0	1	0
39	M	51-60	2012	CHOP	Auto	1	0	0
40	M	41-50	2010	CHOP	Auto	1	1	0
41	M	51-60	2011	CHOP	Auto	0	0	1
42	M	51-60	2006	AOP + Mega CHOP + ESHAP	Auto	0	0	1
43	M	51-60	2006	AOP + Mega CHOP + ESHAP	0	1	1	1
44	F	41-50	2006	MTX + Mega CHOP	0	1	1	1

Legend to abbreviations used in Table 6: ABVD, doxorubicin, bleomycin, vinblastine, dacarbazine ; ACVBP, doxorubicin, cyclophosphamide, vindesine, bleomycin, prednisone; AOP, doxorubicin, vincristine, prednisolone ; CHEP, cyclophosphamide, doxorubicin, etoposide, prednisone; CHOEP, cyclophosphamide, doxorubicin, vincristine, etoposide, prednisone; CHOP, cyclophosphamide, doxorubicin, vincristine, prednisone; CVP, cyclophosphamide, etoposide, prednisone; ESHAP, etoposide, methylprednisolone, cytarabine, platinum; ICE, ifosfamide, carboplatin, etoposide; MTX, methotrexate; SCT, stem cell transplantation.

Supplementary Tables 7. Primary antibodies for ChIP, ChIP-seq, IHC, flow cytometry and immunoblotting .

ChIP and ChIP-seq		
**Name**	**Catalogue No.**	**Company**
anti-BATF3	AF7437	R&D Systems
anti-H3K27ac	ab4729	Abcam

IHC		
**Name**	**Catalogue No.**	**Company**
anti-CD30	M0751	Dako
anti-IL-2	sc-7896	Santa Cruz
anti-IL-2Rα	125M-16	Cell Marque
anti-IL-2Rβ	ab197934	Abcam
anti-IL-2Rγ	PA5-26461	Invitrogen
anti-BATF3	AF7437	R&D Systems
anti-IL-15	MAB2471	R&D Systems
anti-IL-15Rα	AF247	R&D Systems

Flow Cytometry		
**Name**	**Catalogue No.**	**Company**
anti-IL-2Rα	12-0259-42	Invitrogen
anti-IL-2Rβ	11-1228-42	Invitrogen
anti-IL-2Rγ	PA5-26461	Invitrogen
anti-CD30	11-0309-41	Invitrogen
anti-IL-15Rα	AF247	R&D Systems
Isotype control	12-4714-82	Invitrogen
Isotype control	11-4714-82	Invitrogen
Isotype control	02-6102	Invitrogen
Isotype control	AB-108-C	R&D Systems

Immunoblotting		
**Name**	**Catalogue No.**	**Company**
anti-BATF3	AF7437	R&D Systems
anti-STAT1	9172	Cell Signaling
anti-pSTAT1 (Y701)	9167	Cell Signaling
anti-STAT3	12640	Cell Signaling
anti-pSTAT3 (Y705)	9131	Cell Signaling
anti-STAT5	In-house	
anti-pSTAT5 (Y694)	71-6900	Invitrogen
anti-ERK1/2	4695	Cell Signaling
anti-pERK1/2 (T202/Y204)	4370	Cell Signaling
anti-PARP1	9532	Cell Signaling
anti-β-ACTIN	4967	Cell Signaling
anti-β-TUBULIN	2146	Cell Signaling
anti-GAPDH	2275-PC-100	Trevigen

Supplementary Tables 8. Primers for quantitative RT-PCR, semiquantitative RT-PCR and ChIP analyses; oligonucleotides for EMSA analyses and gRNA oligonucleotides for CRISPR/Cas9 genome editing.

Primers for Quantitative RT-PCR

Name	5'-sequence-3'	Product size (bp)
HPRT F	TGACCTTGATTTATTTTGCATACC	102
HPRT R	CGAGCAAGACGTTCAGTCCT	
IL2 F	TGGAACTAAAGGGATCTGAAACAAC	121
IL2 R	CAAGTCAGTGTTGAGATGATGCTT	
IL2RA F	AAGCTCTGCCACTCGGAAC	157
IL2RA R	GGCTTCATTTTCCCATGGTGG	
IL2RB F	GCTTTTAGTCTTTGCGGCCC	130
IL2RB R	GAAGGAAGCCCTGGCTGAG	
IL2RG F	ATTTCTGGCTGGAACGGACG	115
IL2RG R	GCCAGTCCCTTAGACACACC	
IL15 F	TCAATCTATGCATATTGATGCTACTTTA	110
IL15 R	TCAAGTGAAATAACTTGTAACTCCAAGA	
IL15RA F	ABI Taqman # Hs00542604_m1	82
IL15RA R	ABI Taqman # Hs00542604_m1	

Primers for Semiquantitative RT-PCR

Name	5'-sequence-3'	Product size (bp)
BATF3 F	CAGCGTCCTGCAGAGGAG	383
BATF3 R	GCTGGGCAGAGGAGTGTC	
GAPDH F	ATGCTGGCGCTGAGTAC	258
GAPDH R	TGAGTCCTTCCACGATAC	

Primers for ChIP

Gene	Acc. Nr.	Name	5'-sequence-3'	Efficiency (%)
IL2RA	GRCh37:10:6054760-6104253:-1	IL2RA_ChIP_S	ATGGCAAGGGTTTATGAGGACAT	90.9
		IL2RA_ChIP_R	CCACATCCCAGAGCCCAGTAAT	
IL2RB	GRCh37:22:37521878-37546030:-1	IL2RB_ChIP_S	GGGTGTTGGTGGTTTGGCTG	81.5
		IL2RB_ChIP_R	ATCCTACCCCTGCCTGCCTA	
TNFRSF8 (*CD30*)	GRCh37:1:12123434-12204264:1	CD30_ChIP_S	GCTGGTGGGGAGGGACTTTT	100
		CD30_ChIP_R	CCTGACCCAGAACAGCTGCT	
Reference_Chr4	GRCh37:4:58100351-58100438	Ref_Ch4_S	TGCAGGGGCAAGCATATTCA	101.4
		Ref_Ch4_R	GGAGATAAAGCTGGGCGACA	

Oligonucleotides for EMSA

Name	5'-sequence-3'
IL2RA/AP-1 - sense	AGCTGTAATCCCAG**TTACTCA**GGAGGCTGAG
IL2RA/AP-1 - antisense	AGCTCTCAGCCTCC**TGAGTAA**CTGGGATTAC
IL2RB/AP-1 - sense	AGCTGAGGAGCAGG**TGAGTCA**AGAGATGTGG
IL2RB/AP-1 - antisense	AGCTCCACATCTCT**TGACTCA**CCTGCTCCTC

gRNAs for CRISPR/Cas9 Genome Editing

Name	5'-sequence-3'
BATF3 gRNA Oligo1	CACCGGCAGCCGCAGCAGCAGGTAG
BATF3 gRNA Oligo2	AAACCTACCTGCTGCTGCGGCTGCC
Non-targeting gRNA Oligo1	CACCGGTGAACCGCATCGAGCTGAA
Non-targeting gRNA Oligo2	AAACTTCAGCTCGATGCGGTTCACC

Supplementary Figure 1

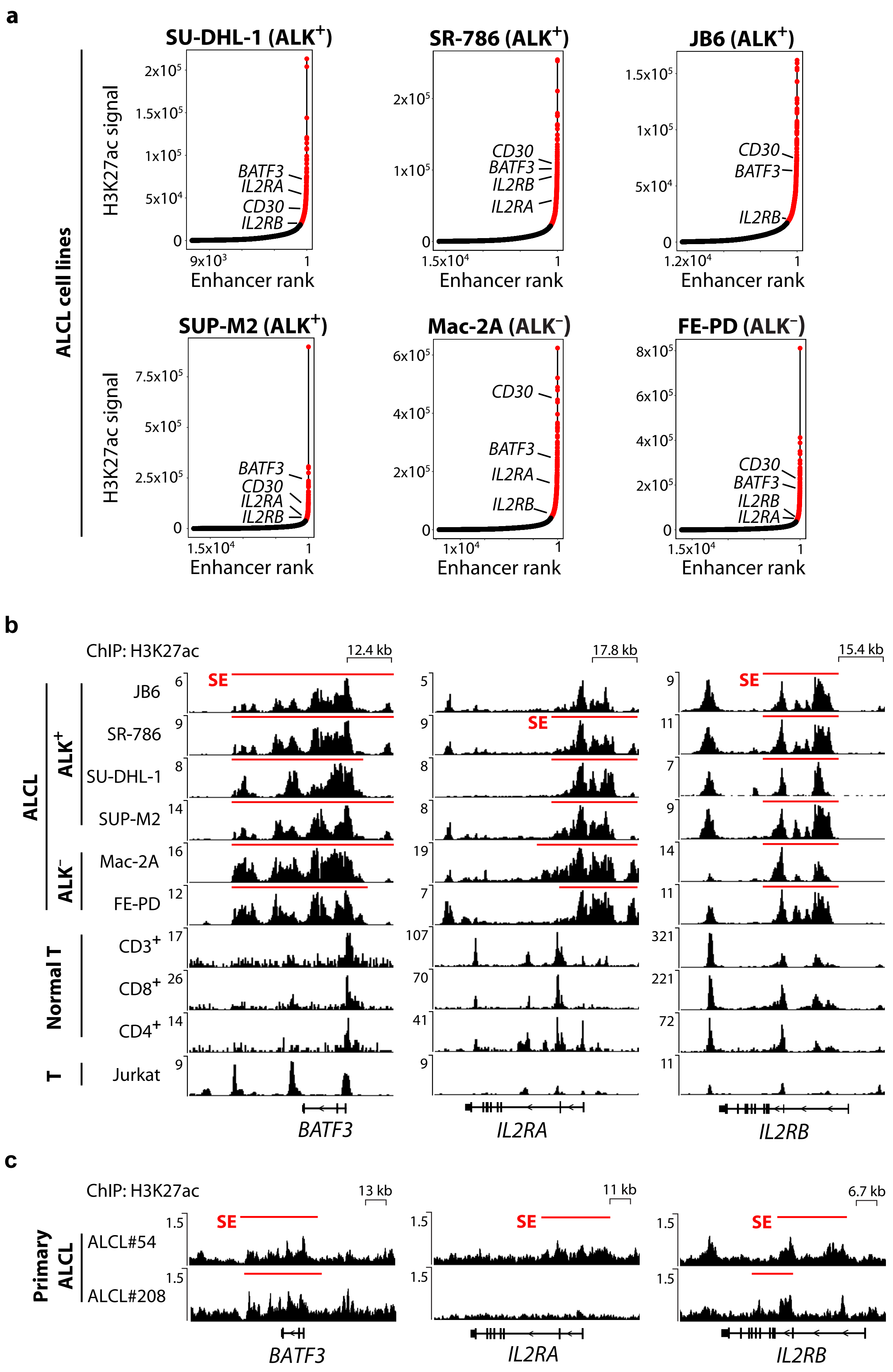

Supplementary Fig. 1: *BATF3*, *IL2RA* and *IL2RB* loci are highly H3K27 acetylated in ALCL but not in normal T cells.

a Enhancers were ranked based on increasing H3K27ac signals in 6 additional ALCL cell lines SU-DHL-1 (ALK^{+}), SR-786 (ALK^{+}), JB6 (ALK^{+}), SUP-M2 (ALK^{+}), Mac-2A (ALK^{-}) and FE-PD(ALK^{-}). Genes within SEs are marked red. **b** ChIP-seq binding profile for H3K27ac at the *BATF3*, *IL2RA* and *IL2RB* loci in indicated 6 ALCL cell lines (4 ALCL, ALK^{+} and 2 ALCL, ALK^{-}), 3 normal T-cell subsets (Normal T: CD3^{+}, CD4^{+} and CD8^{+}) and T-cell leukemia-derived Jurkat control cell line (T) with indicated SE regions. ChIP-seq of H3K27ac and input DNA of normal T-cell subsets (CD3^{+}: GSM1058764, GSM1058789; CD4^{+}: GSM772835, GSM772916; CD8^{+}: GSM1102781, GSM1102806) and Jurkat cell line (GSM1296384, GSM569086) are acquired from previously published datasets. **c** ChIP-seq binding profile for H3K27ac at the *BATF3*, *IL2RA* and *IL2RB* loci in primary ALCL patient samples (#54 and #208) with SE regions indicated.

Supplementary Figure 2

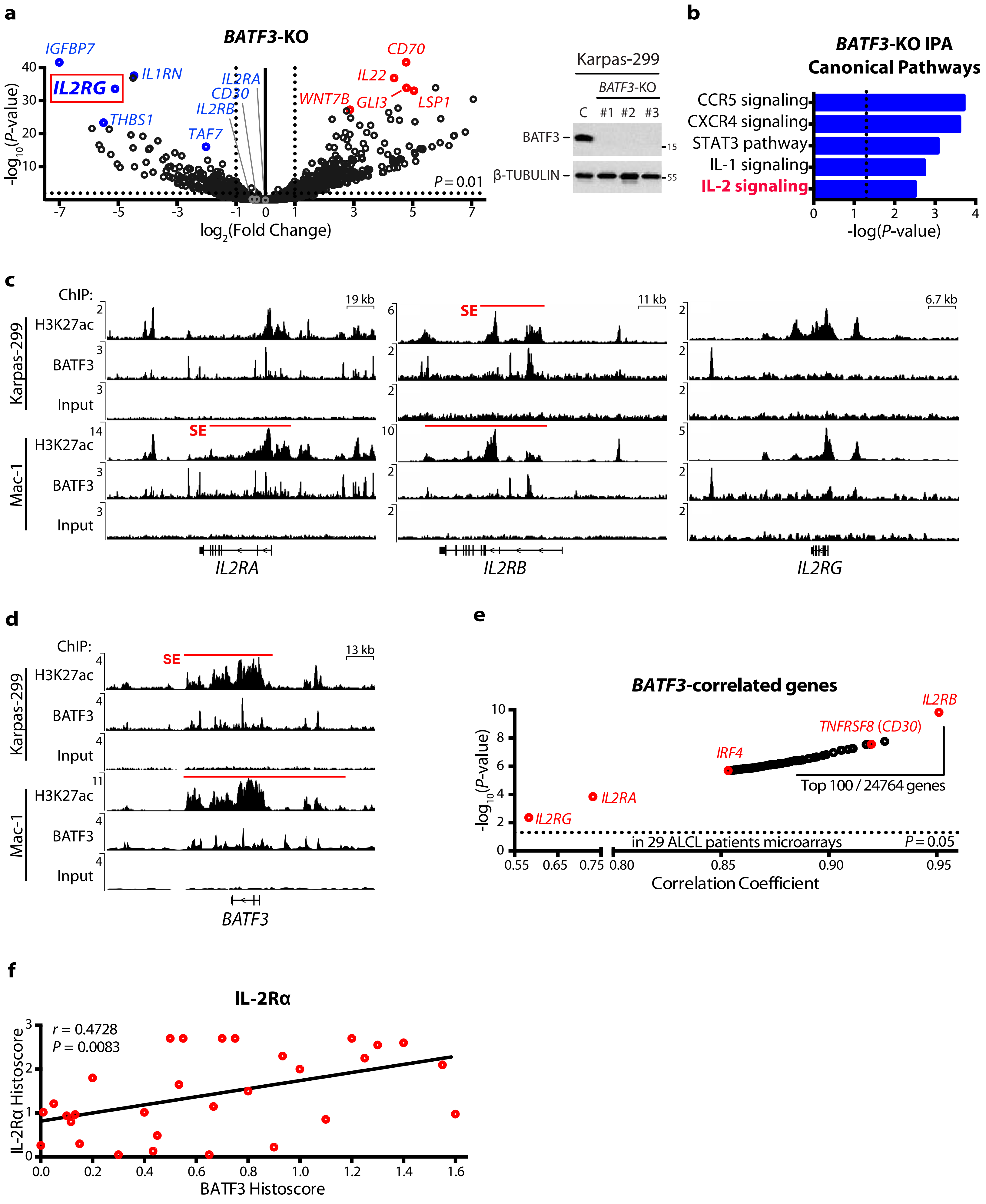

Supplementary Fig. 2: *BATF3* expression correlates with IL-2 signaling in ALCL.

a Volcano plot of differentially expressed genes analyzed by RNA-seq between CRISPR/Cas9-mediated *BATF3*-KO and non-targeting gRNA control Karpas-299 (ALK^+) ALCL cells. Immunoblots confirm the loss of BATF3 expression in three different single clones used for RNA-seq. Blots shown are representative of three independent experiments with similar results. **b** Identification of enriched pathways using IPA 2020 canonical pathway analysis in genes deregulated by CRISPR/Cas9-mediated *BATF3*-KO in Karpas-299 ALCL cells. **c** ChIP-seq tracks at the *IL2RA*, *IL2RB*, *IL2RG* and **d** *BATF3* loci for BATF3 and H3K27ac in indicated ALCL cell lines. **e** Analysis of *BATF3*-correlated genes in a previously published microarray dataset of 29 ALCL patients (GSE19069). Pearson correlation; two-tailed *P*-value based on t-distribution. **f** Correlation between BATF3 and IL-2Rα expression determined by IHC of ALCL tissue specimens (n = 63: 21 ALCL, ALK^+, 21 ALCL, ALK^- and 21 pcALCL). *P* value was determined by two-tailed Pearson correlation, r = 0.4728.

Supplementary Figure 3

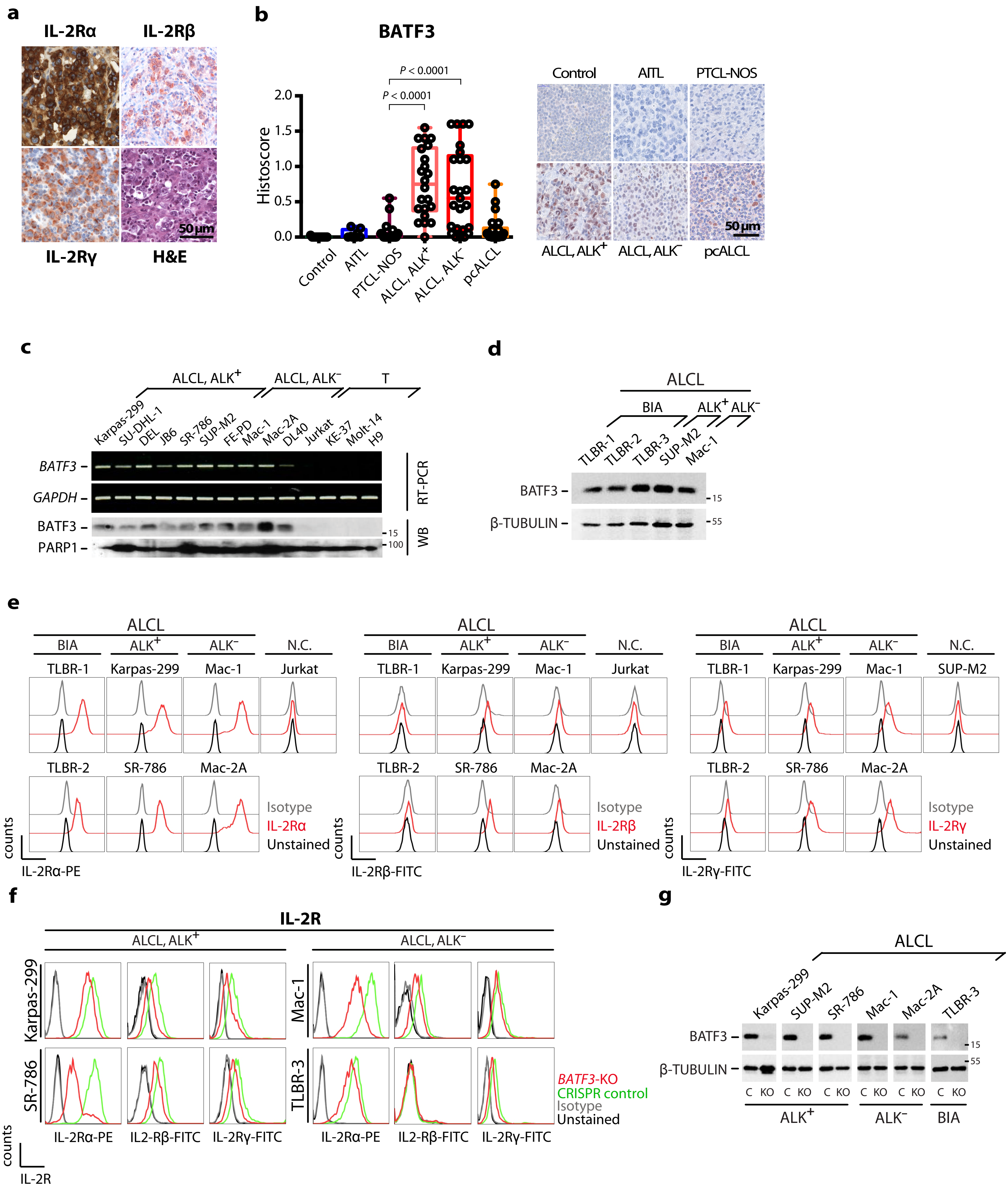

Supplementary Figure 3

h

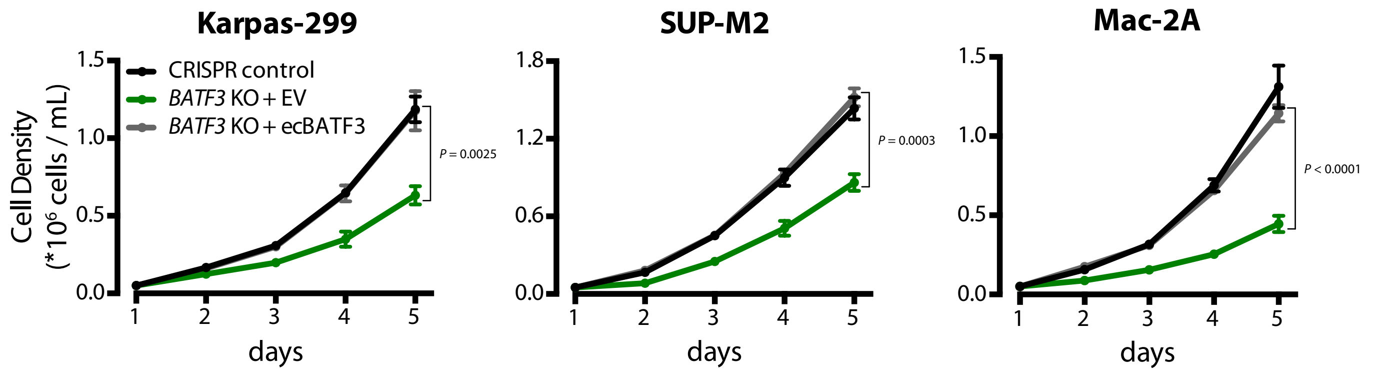

i

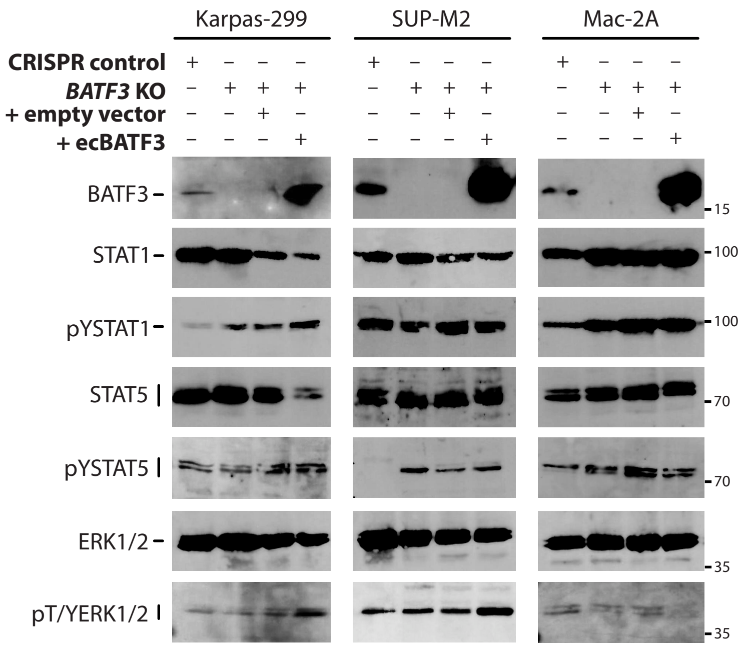

j

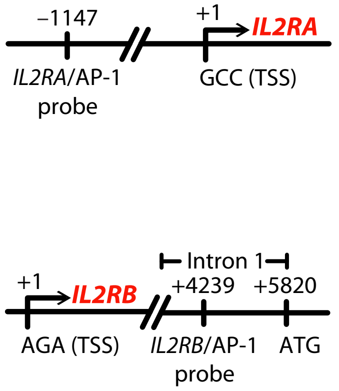

Supplementary Fig. 3: High-level expression of IL-2R subunits in ALCL, mirroring BATF3 expression.

a Representative IHC images of the respective IL-2R subunits and H&E staining, as indicated, in 22 ALCL, ALK^{+} tissue samples measured. **b** IHC quantification and representative IHC images of BATF3 protein expression in an FFPE mature T-NHL TMA (reactive lymph node control, n = 11; AITL, n = 8; PTCL-NOS, n = 23; ALCL, ALK^{+}, n = 22; ALCL, ALK^{-}, n = 23) and pcALCL (n = 24) specimens. *P* values were determined by two-tailed unpaired Student's *t* test. All box-whisker plots represent the median (central line), 25th–75th percentile (bounds of the box) and minimum–maximum (whiskers). **c** Semiquantitative RT-PCR and immunoblot analysis of BATF3 expression in a cell line panel consisting of 10 ALCL cell lines (6 ALCL, ALK^{+} and 4 ALCL, ALK^{-}) and 4 T-cell leukemia-derived control cell lines. *GAPDH* and PARP1 expression serve as control for RT-PCR and immunoblotting, respectively. **d** Immunoblot analysis of BATF3 protein in indicated BIA-ALCL, ALCL, ALK^{+} and ALCL, ALK^{-} cell lines. β-TUBULIN protein expression serves as control. **e** Cell surface expression of IL-2Rα, IL-2Rβ and IL-2Rγ in indicated ALCL and T-cell control cell lines as detected by flow cytometry. Red, IL-2R subunit stained cells; grey, isotype control; black, unstained cells. N.C., negative control. **f** Cell surface expression of IL-2Rα, IL-2Rβ and IL-2Rγ in CRISPR/Cas9-mediated *BATF3*-KO and CRISPR control ALCL cell lines, as indicated. Respective specific staining of the indicated proteins in *BATF3*-KO cells (red) and CRISPR control cells (green); isotype control (grey); unstained cells (black). **g** Immunoblot probing for BATF3 in CRISPR/Cas9-control (C) or *BATF3*-KO (KO) cells of 6 ALCL cell lines. β-TUBULIN serves as control. **h** Growth curves of CRISPR control and *BATF3*-KO cells transfected with either empty vector (EV) or ectopic BATF3 (ecBATF3) in Karpas-299 (ALK^{+}), SUP-M2 (ALK^{+}) and Mac-2A (ALK^{-}) ALCL cell lines. Cell numbers were determined by using a CASY Cell Counter. Values are means ± SD of biological triplicates. *P* values were determined by two-tailed unpaired Student's *t* test. **i** Immunoblot analysis of BATF3, STAT1, STAT5, ERK1/2 proteins and their phosphorylated (pY, pT/Y) forms in CRISPR control and *BATF3*-KO cells transfected with either empty vector or ectopic BATF3

(ecBATF3) of indicated ALCL cell lines. **j** Schematic diagram of *IL2RA* and *IL2RB* subunit regulatory regions with the indication of EMSA probes used (see Supplementary Table 8 for probe sequences). All blots shown in this figure are representative of two independent experiments with similar results in different cell lines.

Supplementary Figure 4

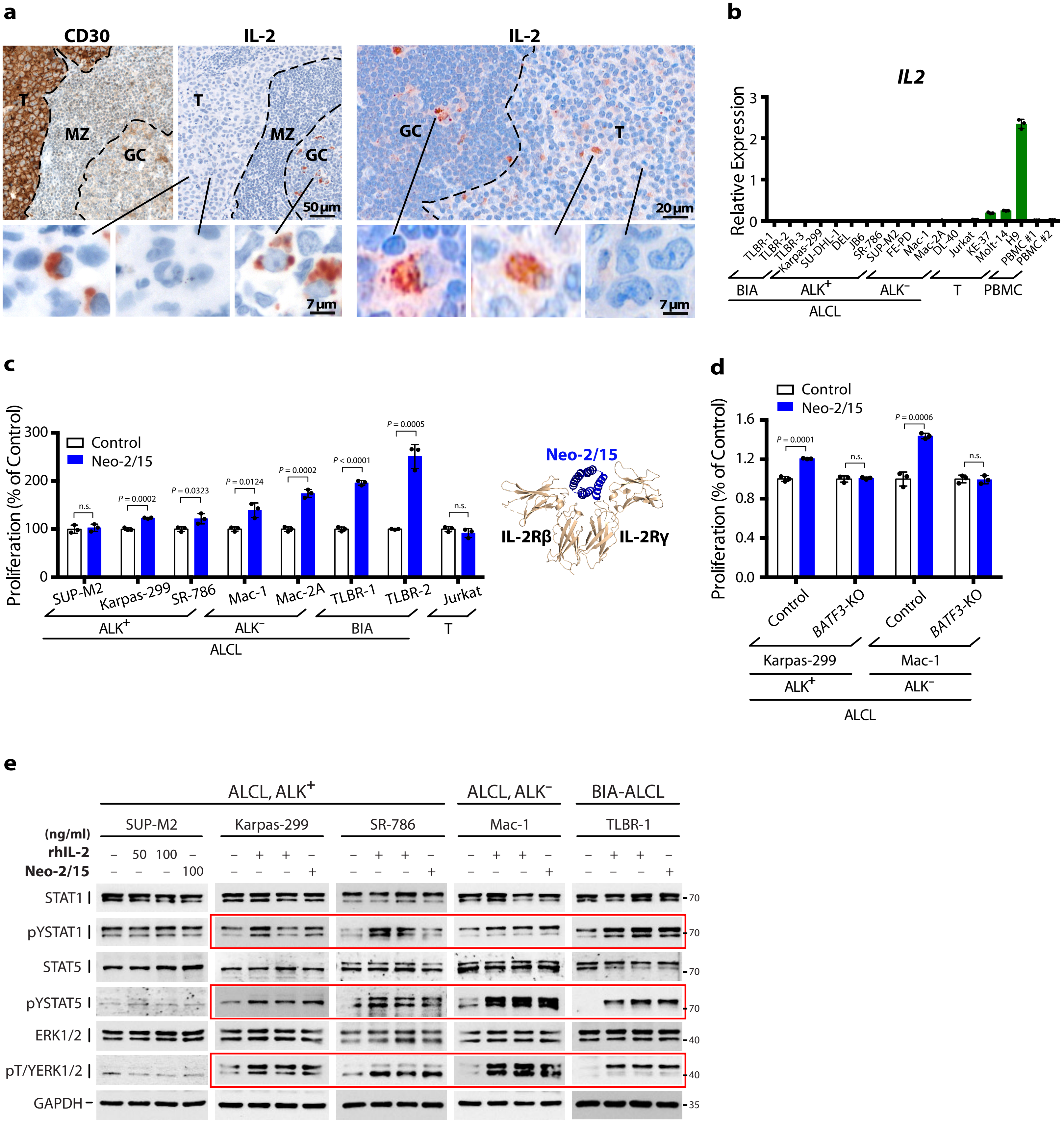

Supplementary Figure 4

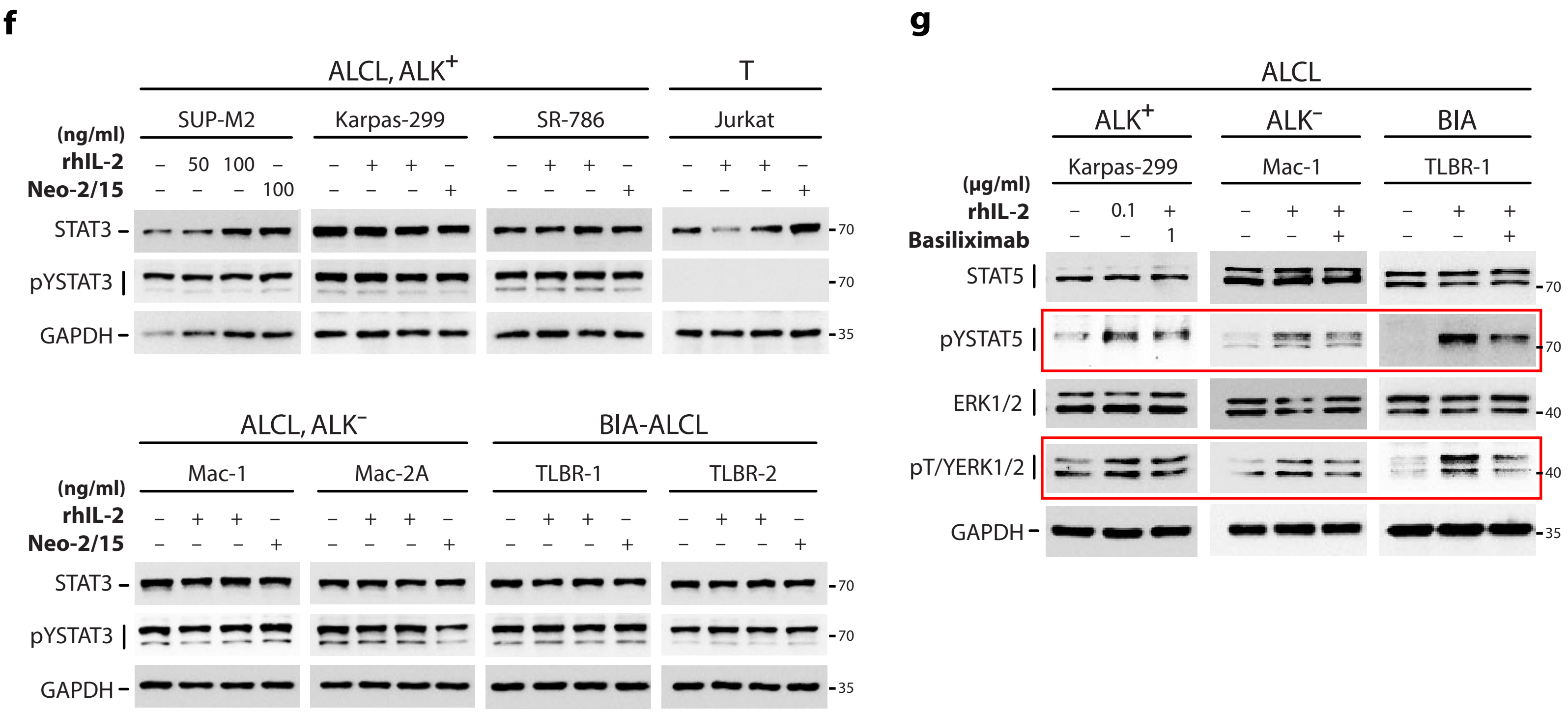

Supplementary Fig. 4: Functional activity of the IL-2/IL-2R system in ALCL.

a Representative IHC images of IL-2 and CD30 from 33 FFPE tissue of primary ALCL, ALK^{+} specimens measured. T, ALCL tumor cells; MZ, lymphatic nodule mantle zone; GC, lymphatic nodule germinal center. Note that CD30 homogeneously marks the ALCL tumor cells, and that IL-2-positive cells are detected in GCs and adjacent to tumor cells. **b** *IL2* mRNA expression in a cell line panel consisting of 13 ALCL cell lines (6 ALCL, ALK^{+}, 4 ALCL, ALK^{-} and 3 BIA-ALCL), 4 T-cell leukemia-derived control cell lines (T) and PBMCs by quantitative RT-PCR. Data are indicated as means ± SD of 3 biological triplicates. **c** Indicated 7 ALCL and Jurkat cell lines were treated with IL-2/IL-15 mimic, Neo-2/15 (100 ng/ml). SUP-M2 (ALK^{+}, IL-2Rγ-negative) and Jurkat (T-cell leukemia-derived, IL-2Rα- and IL-2Rβ-negative) serve as control cell lines. The resazurine assay was used to measure metabolic activity as a marker of cell proliferation. Values are means ± SD of biological triplicates given as a percentage of the untreated control. *P* values were determined by two-tailed unpaired Student's *t* test; n.s., not significant. A structural model of Neo-2/15 that only binds to IL-2Rβγ heterodimer but not IL-2Rα is shown (PDB ID: 6DG5). **d** Abrogation of Neo-2/15-induced proliferation in Karpas-299 (ALK^{+}) and Mac-1 (ALK^{-}) *BATF3*-KO cells compared to CRISPR control. Proliferation was determined by resazurine assay. Data are normalized to untreated control and shown as means ± SD of biological triplicates. *P* values were determined by two-tailed unpaired Student's *t* test. **e** Immunoblot analysis of STAT1, STAT5, ERK1/2 and **f** STAT3 proteins and their phosphorylated (pY, pT/Y) forms in indicated ALCL and T-cell control cell lines stimulated with rhIL-2 (50 or 100 ng/ml), Neo-2/15 (100 ng/ml) or, as a control, PBS (−). GAPDH protein expression serves as control. Blots shown are representative of two independent experiments with similar results in different cell lines. **g** Blockade of IL-2 signal by Basiliximab, the IL-2Rα blocking antibody, in indicated ALCL cell lines is confirmed by immunoblot analysis of phosphorylated STAT5 and ERK1/2 proteins. GAPDH protein expression serves as control. Blots shown are representative of two independent experiments with similar results in different cell lines.

Supplementary Figure 5

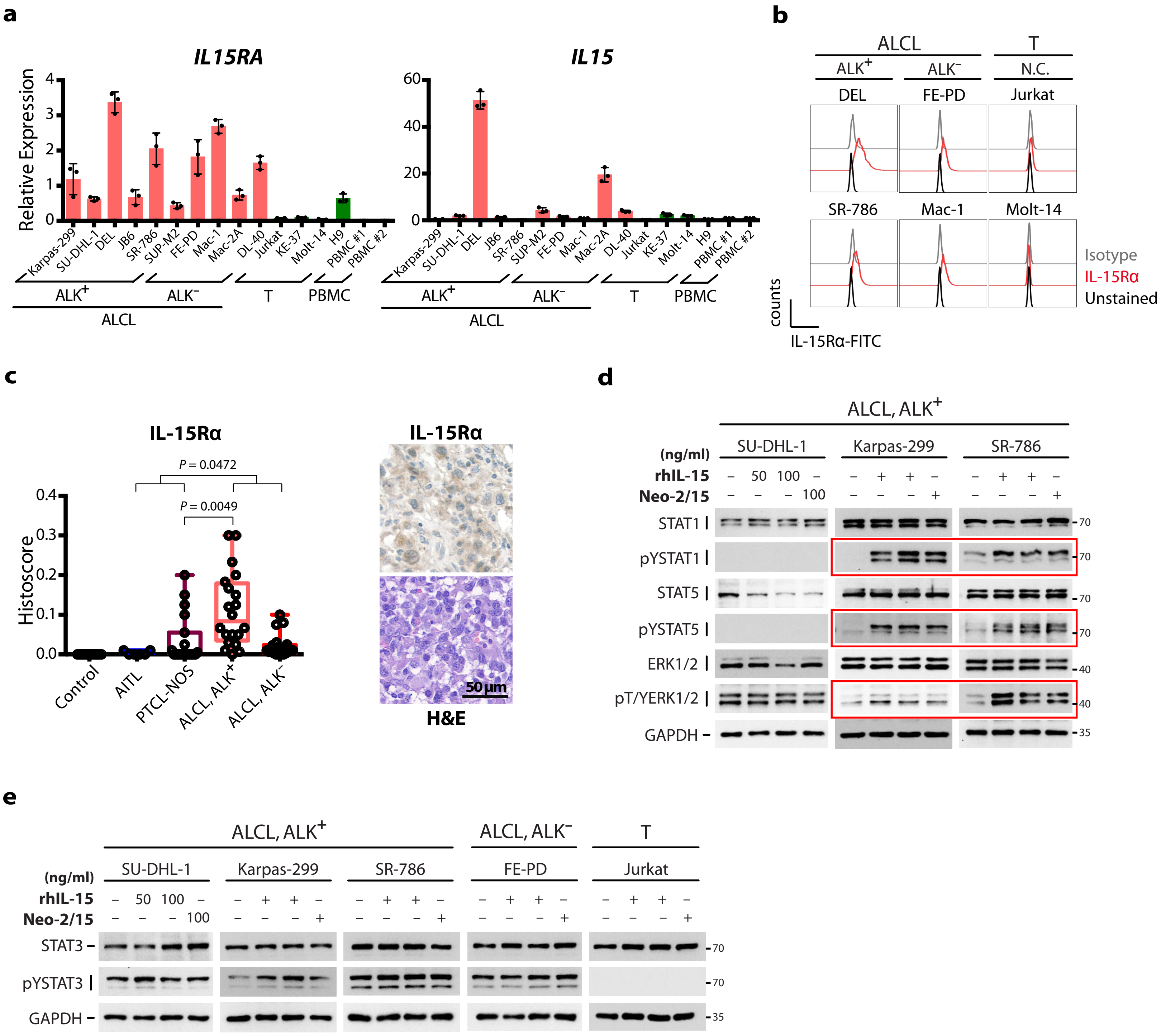

Supplementary Fig. 5: Functional activity of the IL-15/IL-15R system in ALCL.

a *IL15RA* and *IL15* mRNA expression in a cell line panel consisting of 10 ALCL cell lines (6 ALCL, ALK^{+} and 4 ALCL, ALK^{-}), 4 T-cell leukemia-derived control cell lines (T) and PBMCs by quantitative RT-PCR. Data are indicated as means ± SD of biological triplicates. **b** Cell surface expression of IL-15Rα in indicated ALCL and T-cell control cell lines as detected by flow cytometry. Red, IL-15Rα stained cells; grey, isotype control; black, unstained cells. N.C., negative control. **c** IHC quantification of IL-15Rα protein expression in an FFPE mature T-NHL TMA (reactive lymph node control, n = 11; AITL, n = 8; PTCL-NOS, n = 23; ALCL, ALK^{+}, n = 22; ALCL, ALK^{-}, n = 23). *P* values were determined by two-tailed unpaired Student's *t* test. All box-whisker plots represent the median (central line), 25th–75th percentile (bounds of the box) and minimum–maximum (whiskers). Representative IHC and H&E images of ALCL tissue are shown. **d** Immunoblot analysis of STAT1, STAT5, ERK1/2 and **e** STAT3 proteins and their phosphorylated (pY, pT/Y) forms in ALCL and T-cell control cell lines stimulated with rhIL-15 (50 or 100 ng/ml), Neo-2/15 (100 ng/ml) or, as a control, PBS (−). GAPDH protein expression serves as control. Blots shown are representative of two independent experiments with similar results in different cell lines.

Supplementary Figure 6

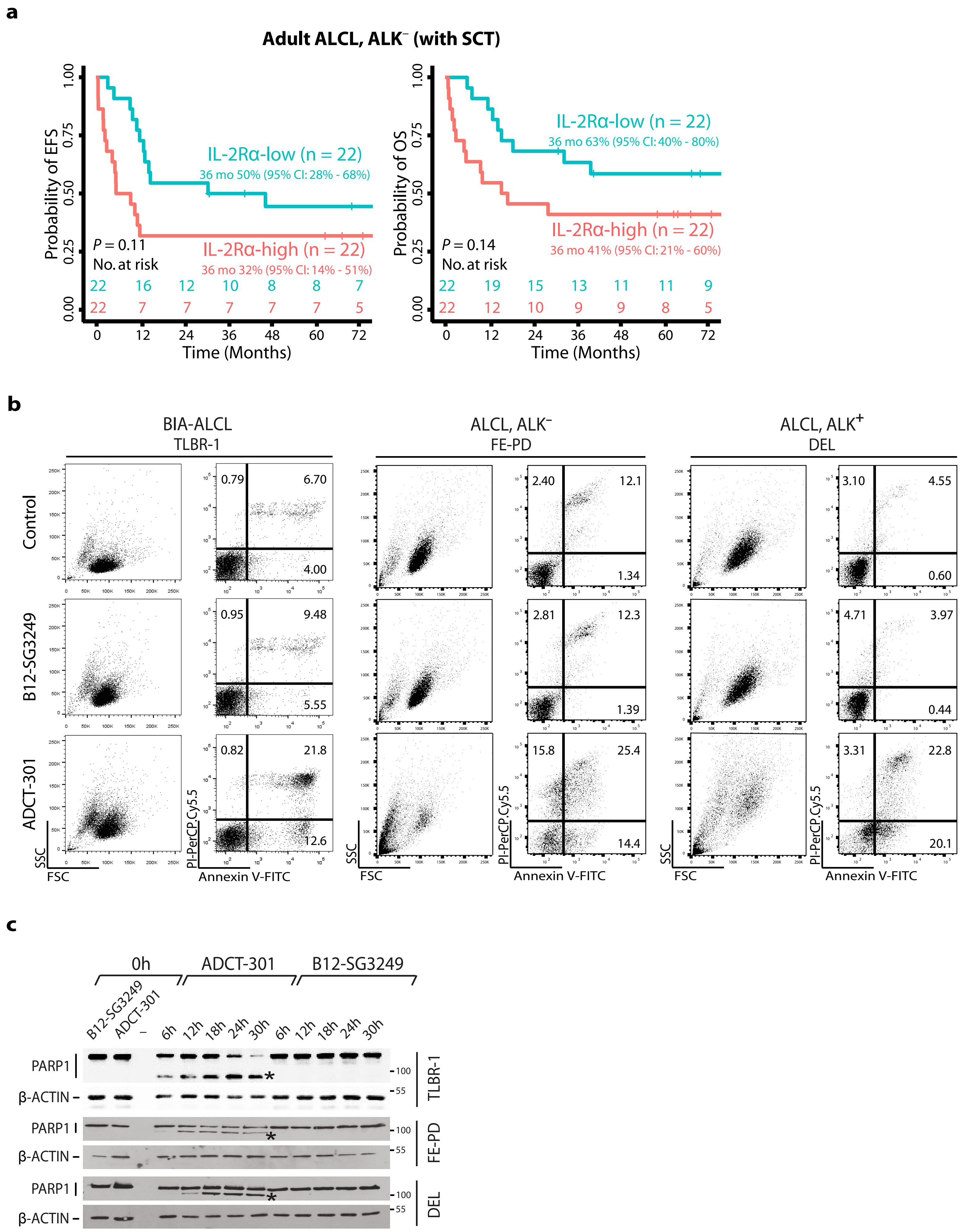

Supplementary Figure 6

d

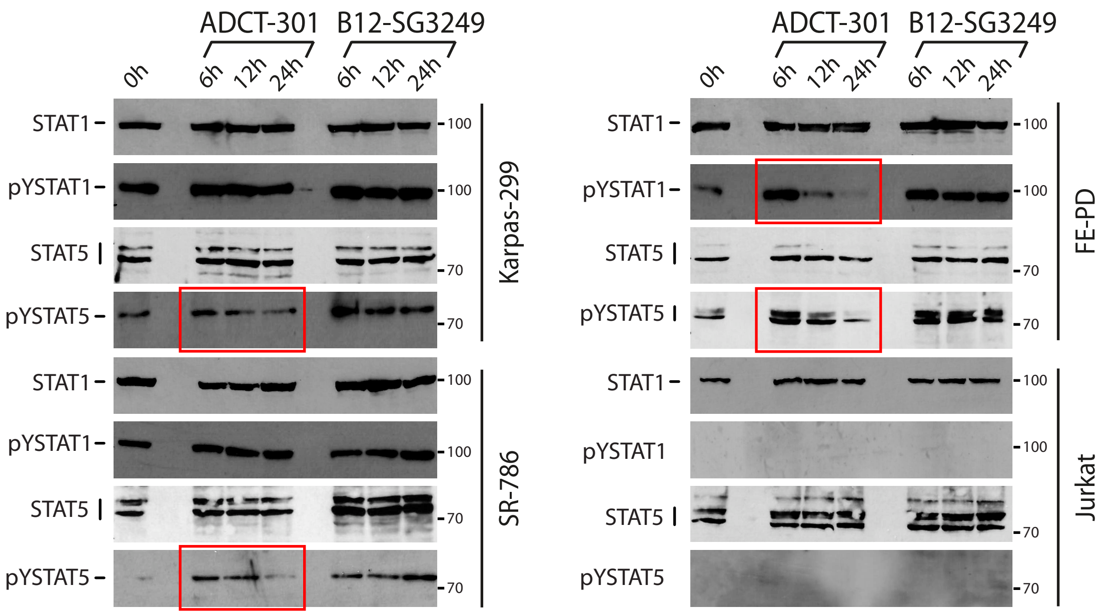

e

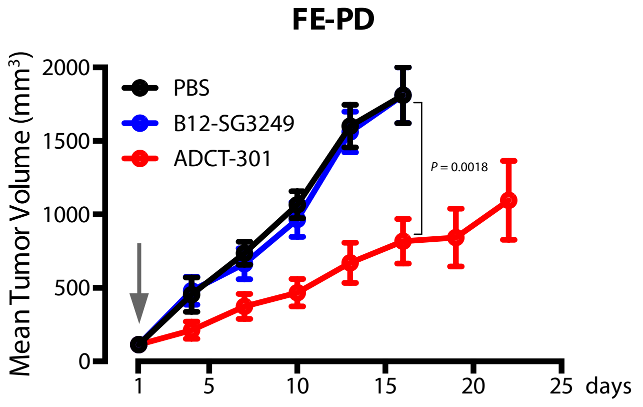

f

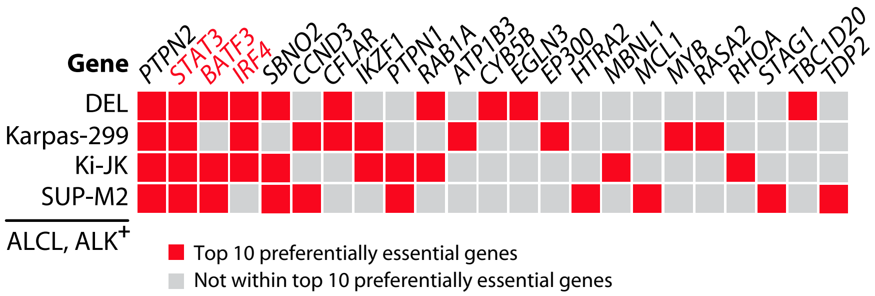

Supplementary Fig. 6: The IL-2Rα-targeting antibody-drug conjugate ADCT-301 exhibits effective killing in ALCL *in vitro* and *in vivo*.

a EFS and OS of adult ALCL, ALK^{-} patients (n = 44) with (n = 10/44) and without (n = 34/44) SCT having low (staining intensity, SI = 0-2) or high (SI = 3) IL-2Rα expression. Kaplan-Meier curves of individual groups were compared using two-tailed log-rank statistics. **b** ADCT-301 induces apoptosis of ALCL cell lines. The ALCL cell lines TLBR-1 (BIA), FE-PD (ALK^{-}) and DEL (ALK^{+}) were left untreated, or treated with 156 ng/ml ADCT-301 or, as a control, ADC B12-SG3249. At 18 (FE-PD) or 24 (TLBR-1 and DEL) hours, cells were analyzed by flow cytometry following Annexin V-FITC/PI staining by flow cytometry. One of three biological replicates is shown. **c** Cells were left untreated (0h), or treated for the indicated times with 156 ng/ml ADCT-301 or ADC B12-SG3249. Thereafter, immunoblot analysis for PARP1 cleavage, indicating apoptotic cell death, and **d** STAT1, STAT5 and their phosphorylated (pY) form was performed. Note that cleaved PARP1 (marked by asterisks) and decreased STAT1 and STAT5 phosphorylation (highlighted by red rectangle) are observed in ADCT-301- but not B12-SG3249-treated cells. β-ACTIN protein expression serves as control. Blots shown are representative of two independent experiments with similar results in different cell lines. **e** *In vivo* anti-tumor efficacy of ADCT-301 in murine xenograft models of FE-PD (ALK^{-}) ALCL cell line. Mice were randomized into three groups to receive a single dose of either vehicle (PBS, n = 4), control ADC (B12-SG3249, 0.5 mg/kg, n = 5), or ADCT-301 (0.5 mg/kg, n = 5) intravenously at day 1 (indicated by an arrow). Tumor volumes were measured over time and are shown as means ± SEM. *P* value was determined by two-tailed unpaired Student's *t* test. two-tailed unpaired Student's *t* test. **f** Top 10 preferentially essential genes in indicated 4 ALCL, ALK^{+} cell lines based on the Achilles CRISPR screens from the DepMap project (depmap.org).
